# Identification and validation of Rab GTPases RAB13 as biomarkers for peritoneal metastasis and immune cell infiltration in colorectal cancer patients

**DOI:** 10.3389/fimmu.2024.1403008

**Published:** 2024-08-13

**Authors:** Fei Lv, Xiaoqi Li, Zhe Wang, Xiaobo Wang, Jing Liu

**Affiliations:** ^1^ Department of Oncology, Shengjing Hospital of China Medical University, Shenyang, Liaoning, China; ^2^ Oncology Department III, People’s Hospital of Liaoning Province, Shenyang, Liaoning, China; ^3^ Department of Digestive Diseases 1, Liaoning Cancer Hospital & Institute, Shenyang, Liaoning, China

**Keywords:** colorectal cancer, peritoneal metastasis, Rab13, macrophage, scRNA-seq

## Abstract

**Background:**

As one of the most common cancer, colorectal cancer (CRC) is with high morbidity and mortality. Peritoneal metastasis (PM) is a fatal state of CRC, and few patients may benefit from traditional therapies. There is a complex interaction between PM and immune cell infiltration. Therefore, we aimed to determine biomarkers associated with colorectal cancer peritoneal metastasis (CRCPM) and their relationship with immune cell infiltration.

**Methods:**

By informatic analysis, differently expressed genes (DEGs) were selected and hub genes were screened out. RAB13, one of the hub genes, was identificated from public databases and validated in CRC tissues. The ESTIMATE, CEBERSORT and TIMER algorithms were applied to analyze the correlation between RAB13 and immune infiltration in CRC. RAB13’s expression in different cells were analyzed at the single-cell level in scRNA-Seq. The Gene Set Enrichment Analysis (GSEA) was performed for RAB13 enrichment and further confirmed. Using oncoPredict algorithm, RAB13’s impact on drug sensitivity was evaluated.

**Results:**

High RAB13 expression was identified in public databases and led to a poor prognosis. RAB13 was found to be positively correlated with the macrophages and other immune cells infiltration and from scRNA-Seq, RAB13 was found to be located in CRC cells and macrophages. GSEA revealed that high RAB13 expression enriched in a various of biological signaling, and oncoPredict algorithm showed that RAB13 expression was correlated with paclitaxel sensitivity.

**Conclusion:**

Our study indicated clinical role of RAB13 in CRC-PM, suggesting its potential as a therapeutic target in the future.

## 1 Introduction

Colorectal cancer (CRC) is with about 2 million new cases and 1 million deaths from the disease worldwide every year (1), making it one of the most common malignancies. Most of CRCs are poorly differentiated and with distant metastasis, and for unresectable patients, more than 1/3 of the patients died of disease progression within 5 years (2). Surgery, chemoradiotherapy, targeted therapy and immuno-therapy are the conventional treatment for CRC, but the effect is limited and the side effects are huge.

Patients with advanced CRC may experience metastasis to different organs, including liver, peritoneum and lung (3). Patients with CRC-PM often mean that they cannot achieve no evidence of disease (NED) through local treatments such as surgery, which is different from liver or lung metastasis. PM is discovered in approximately 13% of CRC cases, most of which are peritoneal and combined with other organs (4). The average survival of CRC-PM patients was shorter than patients with liver-isolated metastasis. Therefore, it is important to explore the pathogenesis of CRC-PM, and to identify new diagnostic markers.

The Ras, Rho, Ran, Arf, and Rab families constitute the small GTPases of the Ras superfamily, which are core regulators of intracellular trafficking signals as scaffold proteins and regulate molecules and downstream effectors (5, 6). One such Rab GTPases, namely RAB13, controls cellular functions that are often altered in cancer, and found to be up-regulated in different kinds of cancers. For example, in ovarian cancer, RAB13 was found as a marker of epithelial-mesenchymal transition (EMT) and could promote metastasis by regulating cytoskeleton and tight junctions (7). However, the role of RAB13 in CRC is still not clear.

As a branch of artificial intelligence, machine learning (ML) has been applied in different fields to analyze massive amounts of data. Several studies described the applicability of ML models to cancer studies (8). ML combines large amount of variables by a computer algorithm, while traditional statistical methods only use selected variables for further calculations, so ML improves the forecasting accuracy (9). Here, we combined bulk RNA-seq with scRNA-seq to investigate the role of RAB13 in CRC-PM and immune cell infiltration.

In this study, we detected abnormal RAB13 expression in CRC-PM. We also demonstrated RAB13 promoted CRC-PM by promoting macrophage infiltration. Therefore, RAB13 is a promising target for CRC-PM immunotherapy.

## 2 Methods and materials

### 2.1 Public data collection

From the National Center of Biotechnology Information Gene Expression Omnibus (NCBI GEO) database, we searched raw gene expression data of the CRC-PM: colorectal AND (cancer OR tumor OR carcinoma) AND peritoneal metastasis. Altogether, 94 datasets were brought into initial search. Mainly inclusion criteria included: (1) The dataset included gene expression profiling data. (2) colorectal cancer samples with or without peritoneal metastasis were involved and at least 3 samples were included each group. By this criteria, two GEO datasets, GSE21510 and GSE87211 were obtained for investigating colorectal cancer peritoneal metastasis hub genes (CRC-PM-Hub Gene).

GEO2R (10) was used to screen DEGs (differentially expressed genes) between samples with or without peritoneal metastasis in the GEO datasets (the cut-off criteria: P-value < 0.05 and absolute log fold-change (FC) > 2) and visualized by volcano plot. Besides, RNA-seq data from TCGA-COAD were also downloaded and analyzed. The RobustRankAggreg (RRA) method was used to integrate and analyze the three datasets (GSE21510 and GSE87211 and TCGA-COAD) to obtain the common DEGs (11). R package ‘VennDiagram’ was used to identify the overlapping genes between the common DEGs and peritoneal metastasis genes obtained from GeneCards database (12).

Gene Ontology (GO) is a standardized language and classification system used to describe gene function and genomic data (13), while Kyoto Encyclopedia of Genes and Genomes (KEGG) is a database resource containing functions and information of biological systems (14). GO and KEGG analysis of the DEGs were performed using WebGestalt (15).

### 2.2 The identification of CRC-PM-hub gene

Using the glmnet package of R (16), Least Absolute Shrinkage and Selection Operator (LASSO) analysis was enforced based on the DEGs. LASSO algorithm are instrumental in mitigating the risk of overfitting and enhancing the accuracy of predictions (17). The model or gene signature was defined as: colorectal cancer peritoneal metastasis risk score (CRC-PM RS) = ∑Exp(i)*coef(i) (i: genes; exp: expression levels; coef: LASSO coefficients). The reliability and stability of the model were proven in the GSE17538 and GSE37892 datasets.

We further validated CRC-PM genes from the LASSO coefficients by two different algorithms in GSE17538 dataset. Random forest is based on multiple decision trees (ensemble), which employed bootstrap aggregation and predictor randomization to attain notable predictive accuracy (18). Support Vector Machine-Recursive Feature Elimination (SVM-RFE) trains a subset of features to reduce the feature set and find the most predictive features (19). In SVM-RFE, the feature genes were the genes corresponding to the minimum point of cross-validation error.

### 2.3 Expression, mutation, methylation and phosphorylation of RAB13 in TCGA-COAD

(1) Expression: TCGA data was collected to analyze the RAB13 mRNA expression level and the survival package was used to estimate the survival rate of TCGA_COAD patients with different RAB13 expression.

(2) Mutation: R package ‘maftools’ was used for the mutation analysis to identify variations of mutated genes in RAB13 high and low expression groups (20, 21).

(3) Methylation: We assessed the survival time of patients with CpG methylation in RAB13 based on the MethSurv database, a web tool to investigate correlations between survival time and CpG methylation patterns (22).

(4) Phosphorylation: The phosphorylation levels of RAB13 were analyzed with the CPTAC database, a central repository for the public dissemination of proteomic sequence dataset (23).

### 2.4 Patients tissue samples and immunohistochemistry

The research was agreed by Ethics Committee of Liaoning Provincial People’s Hospital. Informed consent were signed and complianced in sample collection in all involved patients. A total of 92 CRC tissues were obtained from surgical resection.

Hematoxylin and eosin (H&E) staining and immunohistochemical staining were done to assess RAB13 expression. Immunoreactivity was semi-quantitatively as we described before (24).

### 2.5 Evaluation of immune cell type components

The Estimation of Stromal and Immune Cells (ESTIMATE) algorithm was performed to calculate the ImmuneScore (proportion of immune ingredient) by R package ‘estimate’ (25). By using CIBERSORT (26), we investigated the correlation of RAB13 expression with diverse immune infiltrating cell types. Single-Sample Gene Set Enrichment Analysis (ssGSEA) is to investigate the correlation of tumor-infiltrating immune cells with their associated functional pathways (27). We performed ssGSEA analyze immune signatures using the GSVA package. Immune infiltration from TCGA-COAD was analyzed in Tumor Immune Estimation Resource database (TIMER 2.0).

### 2.6 Analysis of RAB13 in CRC using scRNA-seq data

Single-cell RNA sequencing (scRNA-seq) datasets (GSE136394, GSE161277 and GSE188741) were downloaded from GEO and we analyzed the scRNA-seq data using Seurat R package (28). Cell type annotation was performed by CellMarker database and RAB13’s expression in different cells was visualized by violinplot. Lastly, the R package ‘CellChat’ (29) was employed to analyze cell-to-cell communication networks and interactions.

### 2.7 Gene set enrichment analysis

GSEA is conducted by using GSEA v2.2.2 to identify RAB13 associated gene sets in GSE17538 and GSE37892 (27).

### 2.8 Statistical methods

We analyzed the experimental data by SPSS software (Chicago, USA), who was presented as the mean ± SD. Differences were considered significant when p-values < 0.05.

## 3 Results

### 3.1 Identification of CRC-PM-DEGs in public database

**Figure 1:**
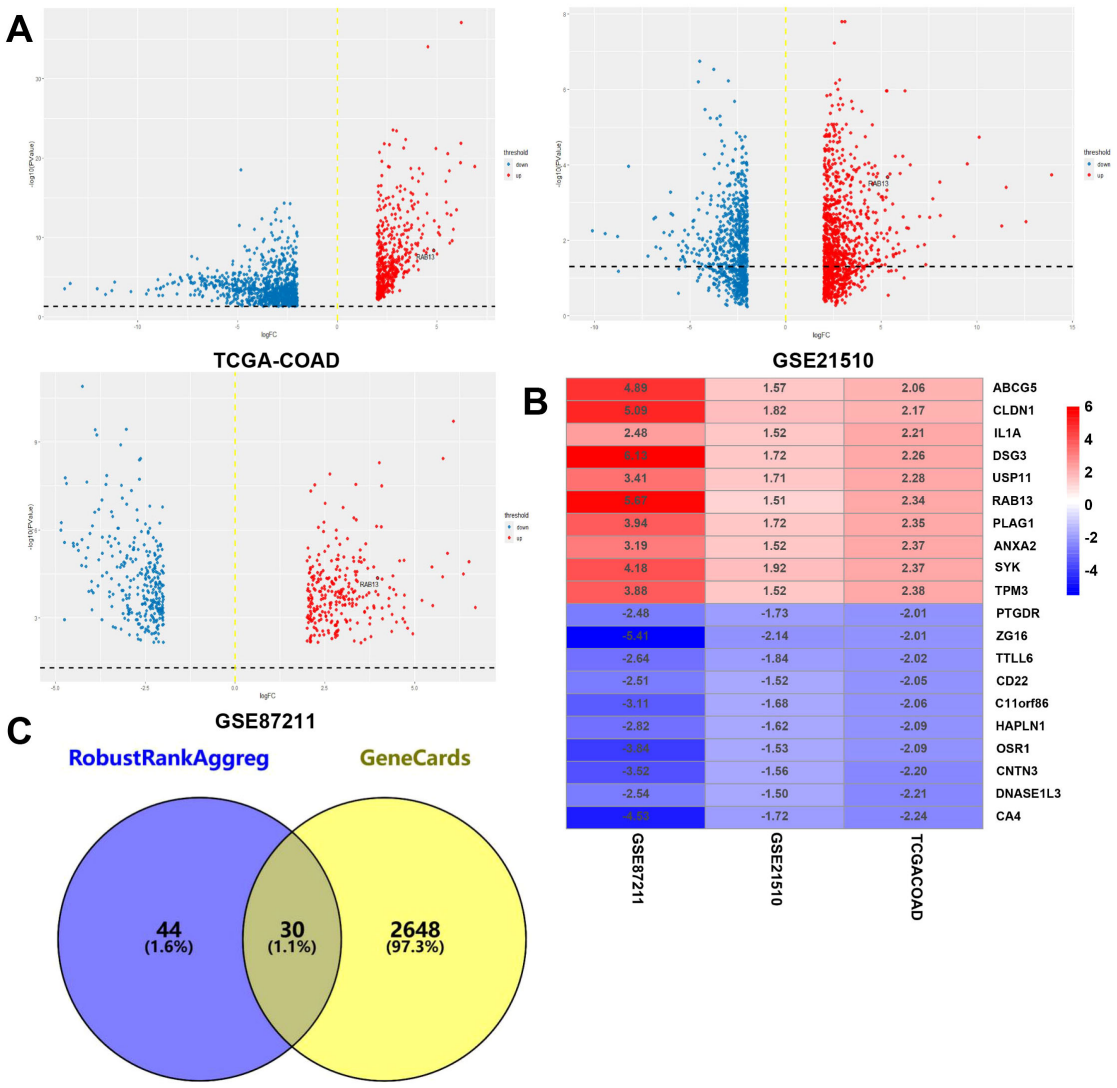
Identification of CRCPM-DEGs in public datasets. **(A)** The volcano plot of mRNA levels from TCGA-COAD, GSE21510) and GSE87211 datasets and the x-axis represented the log2 transformed fold change ratios, while the y-axis was the log10 transformed adjusted p-value. The red colored dots represented the DEGs based on fold change >2. **(B)** Ten upregulated and downregulated DEGs. **(C)** Venn diagram for the overlapping genes of DEGs from RRA algorithm and peritoneal metastasis genes from GeneCards database. A total of 30 overlapping CRCPM-DEGs were obtained.

**Figure 2:**
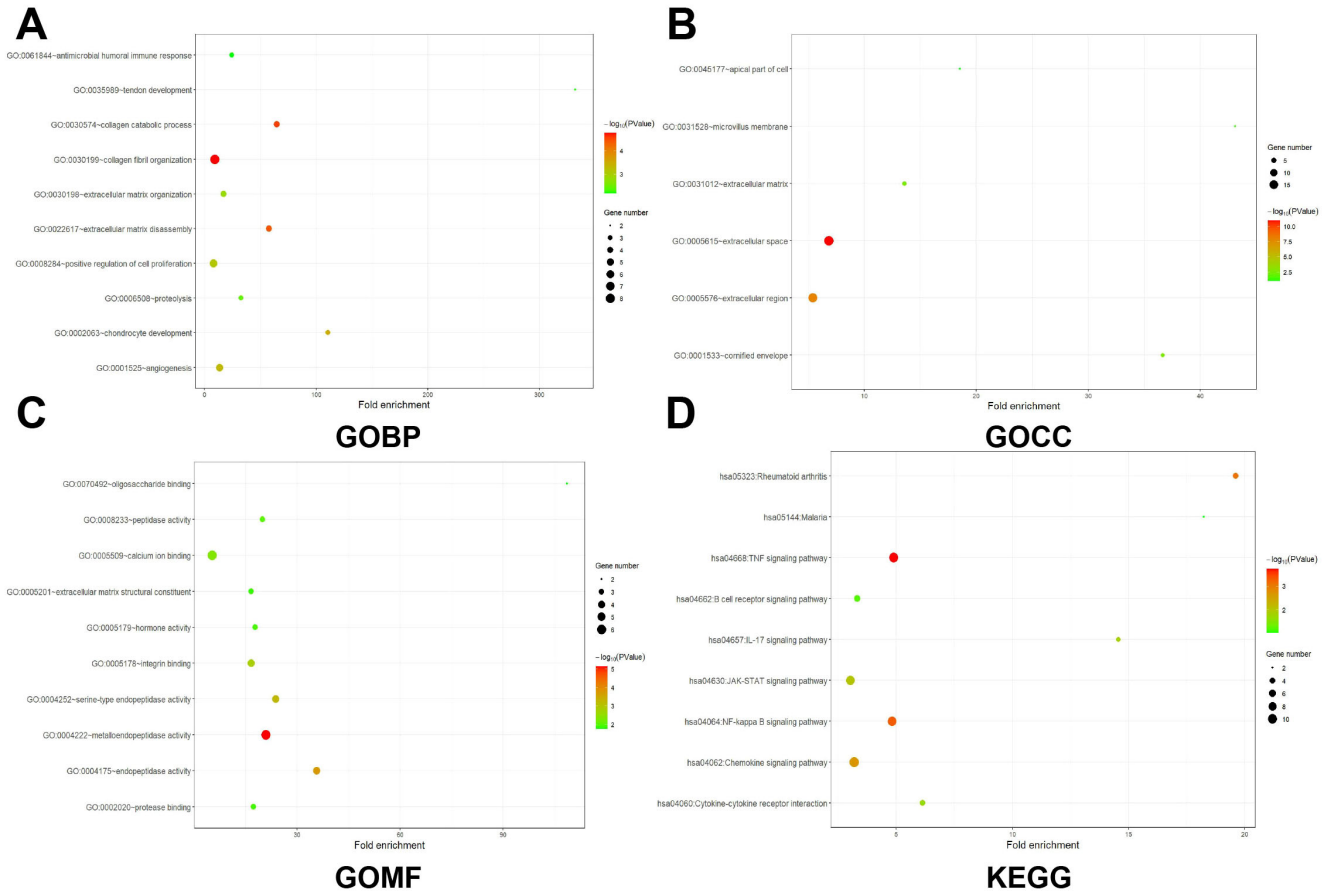
GO and KEGG analysis of CRCPM-DEGs. **(A–C)** Enrichment results of BP (Biological Process, **(A)**, CC [Cellular Component, **(B)**] and MF (Molecular Function, **(C)**. Gene counts were presented by the length of bars and the value of the minus log10 adjusted P value were presented by the gradation of color. **(D)** KEGG analysis results. The gene counts were presented by the dot size and the value of minus log10 P value were presented by the gradation of color.

Following careful screening of the content, the two datasets with the largest sample size (GSE21510 and GSE87211) were obtained. The GSE21510 dataset consisted of 123 CRC and 23 normal samples (30) and the GSE87211 dataset contained 230 CRC and 133 normal samples (31). Besides, we also downloaded gene expression records of 619 CRC patients from TCGA-COAD. We identified 157, 78 and 113 upregulated genes in the GSE21510, GSE87211 and TCGA-COAD datasets, respectively, and visualized them in a volcano plot (**Figure 1A**). The overlapping DEGs were screened by the RRA method and 161 integrated DEGs, comprising 74 upregulated genes and 87 downregulated genes, were identified, as shown in **Supplementary Table 1** and **Figure 1B**. Next, we intersected the 74 upregulated genes with PM genes obtained from the GeneCards database and obtained 30 genes (**Figure 1C**). The GO and KEGG analysis results showed that DEGs may have multiple biological functions: the collagen fibril organization was the most significant biological process (BP) term, extracellular space was the cellular component (CC) term with highest significance, and the most significant (MF) term was metalloendopeptidase activity (**Figures 2A–C**). The KEGG pathway showed that the DEGs were involved in TNF signaling pathways (**Figure 2D**).

### 3.2 Establishment and validation of a signature based on PM-DEGs

**Figure 3:**
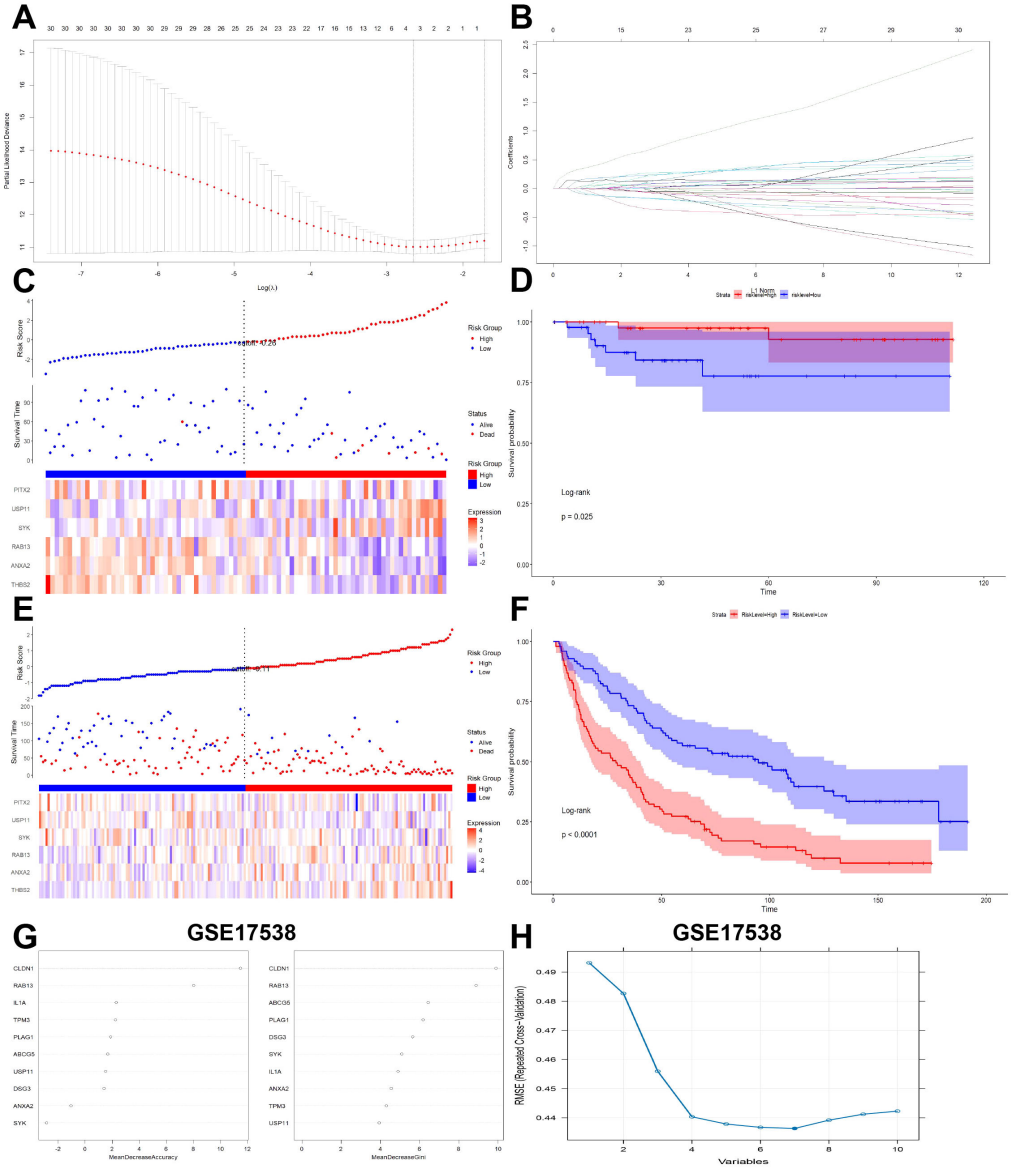
Validation of CRCPM-DEGs in public datasets. **(A, B)** LASSO coefficients profiles **(A)** to determine the number of factors and cross-validation diagram **(B)** for tuning parameter selection in the least absolute shrinkage and selection operator (LASSO) model. From left to right along the x-axis, with the increases of lambda, the compression parameter increased and the absolute value of the coefficient decreased. The number on top were the number of variables with non-zero regression coefficients in the LASSO model. **(C, E)** The distribution of the risk score (upper), overall survival (middle) and heatmap (lower) that made up the signature genes in the training cohort (GSE17538, **(C)**) and validation cohort [GSE37892, **(E)**]. **(D, F)** Kaplan Meier analysis of two risk groups based on signature genes in the training cohort [GSE17538, **(D)**] and validation cohort [GSE37892, **(F)**]. **(G)** Random forests Identify LACBM-DEGs in GSE17538. **(H)** LACBM-DEGs validation by SVM-RFE algorithm selection in GSE17538.

To further validate the prognostic value of CRC-PM-DEGs, LASSO regression was conducted to construct a signature that predict the prognosis of patients in the training cohort, the GSE17538 datasets. Six genes (THBS2, ANXA2, RAB13, SYK, USP11, PITX2) were finally identified to establish the signature (**Figure 3A**). The risk coefficients were calculated from the correlation coefficients of the six hub CRC-PM-DEGs with the following formula (**Figure 3B**): CRC-PM-RS = THBS2*0.27+ANXA2*0.22+RAB13*0.18+SYK*0.06-USP11**0.5-PITX2*0.02.

Based on the CRC-PM-RS, the patients in the GSE17538 dataset were divided into the high- and low-risk subgroups (**Figure 3C**). Survival status scatterplot and Kaplain-Meier survival analyses suggested that patients with high-Risk score had poorer survival than those with low-Risk score (**Figure 3D**). To validate the model, we used the GSE37892 dataset as an external validation cohort. The analysis showed a statistically significant difference in overall survival between the low- and high-risk groups (**Figures 3E–F**).

We next integrated another two different algorithms in GSE17538 dataset to increase diagnostic effectiveness while reducing noise information. By random forest algorithm, the most valuable top 2 genes in CRC-PM was CLDN1 and RAB13 (**Figure 3G**). In addition, the SVM-RFE results show that the best prediction performance can be obtained by selecting 7 feature variables (RAB13, CLDN1, ANXA2, SYK, TPM3, IL1A, USP11, **Figure 3H**). In the CRC-PM-RS formula, RAB13 has a relative high coefficient, which also rank at the front in random forest and SVM-RFE algorithms, so we defined RAB13 as the CRC-PM hub gene for further analysis.

### 3.3 Expression, prognosis, mutation, methylation and phosphorylation of RAB13 in TCGA-COAD

**Figure 4:**
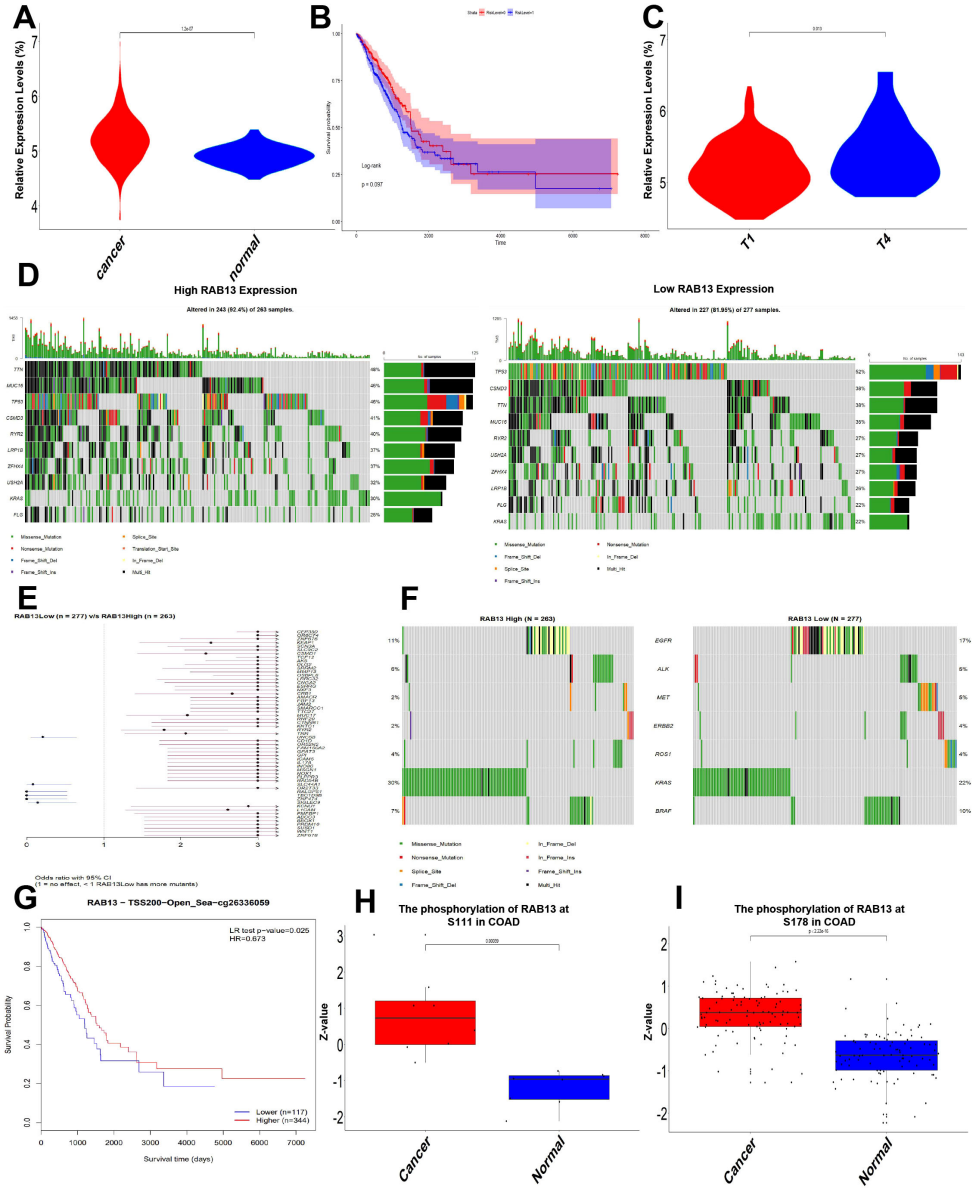
Expression, mutation, methylation and phosphorylation of RAB13 in TCGA-COAD. **(A)** Data from TCGA-COAD dataset showed that compared with para-cancerous tissues, RAB13 mRNA was up-regulated in lung cancer tissues. **(B)** Kaplan-Meier analysis of the relationship between RAB13 expression and OS in TCGA-COAD dataset. **(C)** The expression of RAB13 correlated with T stage in TCGA-COAD dataset. **(D)** Differential somatic mutations were found in COAD with high and low RAB13 expression. **(E)** Forest plot showed that COAD patients in high RAB13 group had more oncogenic mutation. **(F)** Mutations frequencies of therapy target of COAD in RAB13 high or low group. **(G)** Prognostic value of methylation site RAB13-cg26336059 in COAD. **(H, I)** Expression of phosphorylation site of RAB13 at S111 and S178 was analyzed in COAD.

To further understanding the biological function of RAB13, we employed TCGA-COAD to evaluate RAB13 expression levels. It revealed that RAB13 mRNA level was significantly elevated in CRC tissues (**Figure 4A**). Kaplan-Meier analysis showed that high expression of RAB13 has a tendency to prolong survival, but there is no statistical significance (p = 0.09, **Figure 4B**). Expression of RAB13 was also correlated with T stage in TCGA-COAD (**Figure 4C**). CRC is a kind of severe cancer with high heterogeneity and genetic factors (32), so we explored the association between RAB13 expression and somatic mutations in TCGA-COAD by maftools in R. The results showed that somatic mutations differed between RAB13 high and low groups. For example, as shown in **Figure 4D**, 52% of TP53 mutation, which was with the highest mutation frequency, was observed in RAB13 low group, while 46% in RAB13 high group. Pathogenic mutations, such as CTNNB1 (33) are more common in the RAB13 high expression group (**Figure 4E**). Treatment approaches discovered driver events in colorectal cancer, such as mutations in EGFR, led to profound improvements in clinical outcomes (34). The difference of the driver events between RAB13 high and low groups was also explored. As shown in **Figure 4F**, EGFR mutation was higher in RAB13 low group. That means targeted therapy may be more effective in patients with low RAB13 expression.

DNA methylation is an important epigenetic characteristic, and DNA methylation levels of RAB13 with the prognostic value of each single CpG were investigated using the MethSurv tool. One CpG site’s methylation level, namely cg26336059, was strongly associated with prognosis (**Figure 4G**). In the CPTAC dataset, we also found that the phosphorylation levels of S111 and S178 of RAB13 CRC were increased (**Figures 4H, I**).

To further understanding the expression of the CRC-PM hub gene, RAB13, the expression of RAB13 was detected in 92 CRC tissues by immunohistochemistry. The expression of RAB13 was upregulated in CRC tissues for 64.1% of the patients (59/92), and RAB13 was mostly expressed in the cytoplasm (**Supplementary Figures 1A, B**).

### 3.4 RAB13 correlated with tumor immunity in CRC

**Figure 5:**
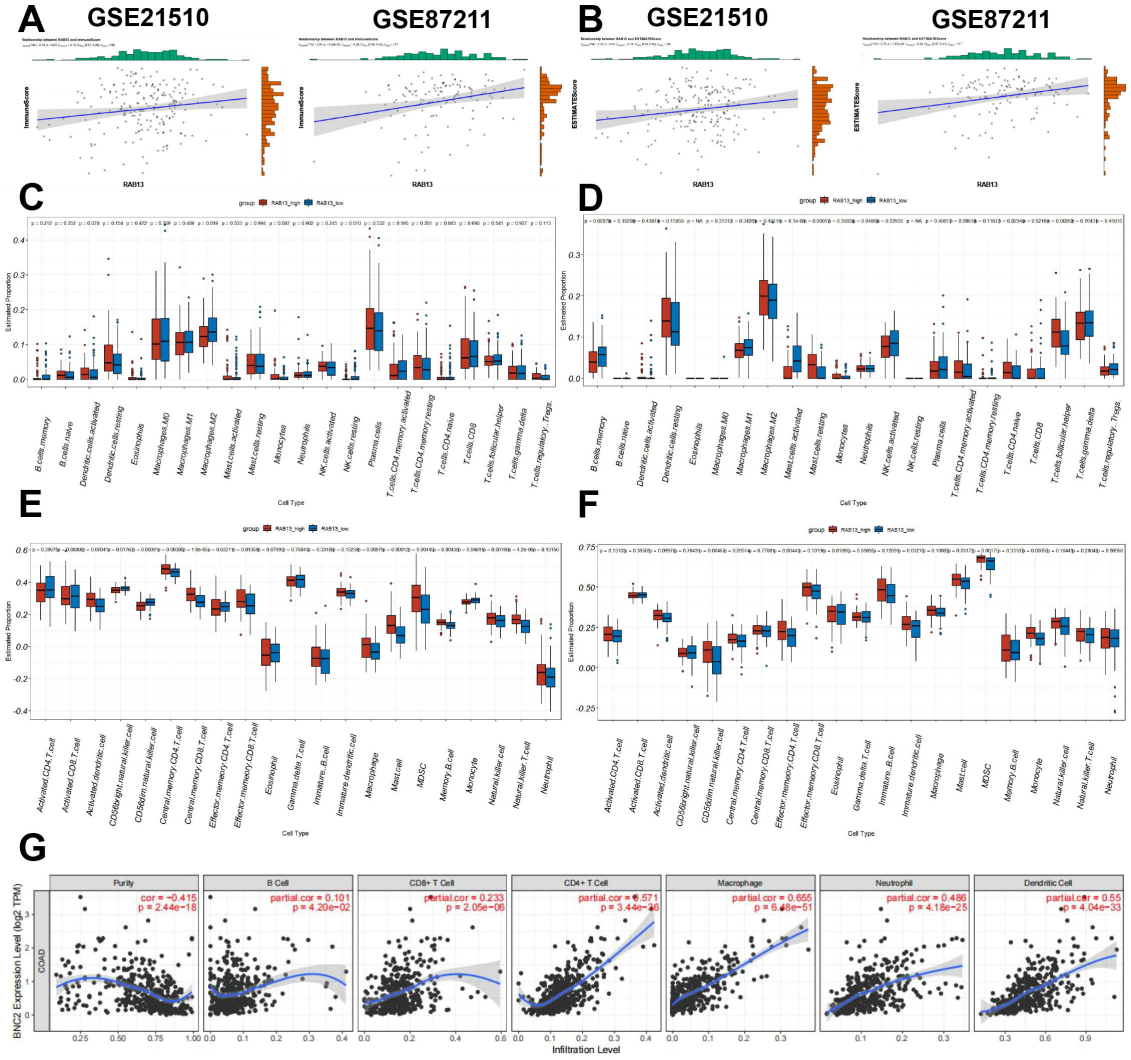
RAB13 correlated with tumor immunity in COAD. **(A, B)** The comparison of ImmuneScore **(A)** and ESTIMATEScore **(B)** between the high- and low- RAB13 groups in the GSE21510 and GSE87211 cohorts by ESTIMATE algorithm. **(C, D)** Comparison of tumor-infiltrating immune cells between the high- and low- RAB13 groups in the GSE21510 and GSE87211 cohorts by CIBERSORT algorithm. **(E, F)** Comparison of tumor-infiltrating immune cells between the high- and low- RAB13 groups in the GSE21510 and GSE87211 cohorts by ssGSEA algorithm. **(G)** Correlations between RAB13 expression and tumor-infiltrating immune cells in COAD obtained from the TIMER database.

We next evaluated the association between RAB13 expression and tumor immunity using ESTIMATE algorithm. As shown in **Figures 5A, B**, the RAB13 expression was significantly positively correlated with Immune Score (GSE21510: Pearson’s r = 0.15, P = 0.03, GSE87211: Pearson’s r = 0.26, P = 5.29e-3) and ESTIMATE Score (GSE21510: Pearson’s r = 0.16, P = 0.02, GSE87211: Pearson’s r = 0.24, P = 7.87e-3). The R-value of correlation analysis was among -0.3~0.3, which was marginally statistical significant, so we employed different algorithms to confirm the conclusion. First, we calculated tumor-infiltrating immune cell proportions in the two GEO datasets employing CIBERSORT algorithm. In GSE21510 dataset, M2 macrophages and resting NK cells were decreased in RAB13 high group (**Figure 5C**), while in GSE87211 datasets, memory B cells and mast cells were changed in RAB13 high group (**Figure 5D**). Next, the tumor-infiltrating immune cells in CRC was further analyzed by ssGSEA in the two GEO datasets, which showed significantly positive correlations between RAB13 expression and Monocyto-Macropahges or Mast cell (**Figures 5E, F**). At last, the TIMER database also showed that RAB13 expression was correlated with different immune cells infiltration in TCGA-COAD datasets (**Figure 5G**).

**Figure 6:**
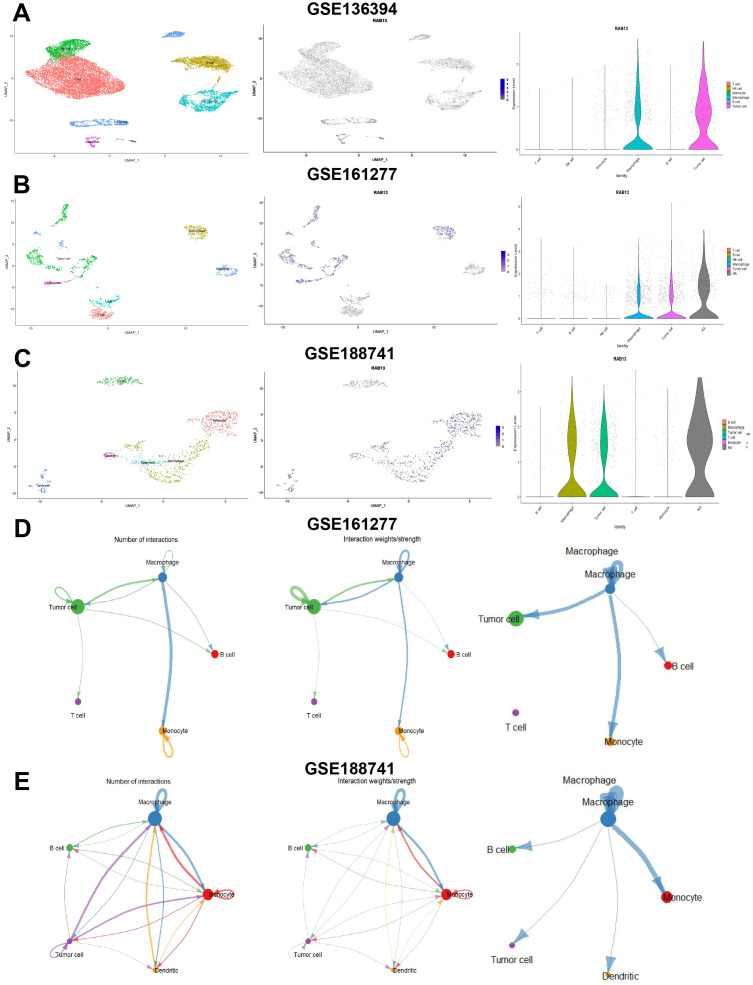
Single cell analysis of RAB13 in COAD. **(A-C)** Left: UMAP plot visualization of all cell subtypes from COAD patients from GSE136394 **(A)**, GSE161277 **(B)** and GSE188741 **(C)** datasets. Middle: UMAP plot visualization of the distribution of RAB13. Right: Violin plot visualization of the distribution of RAB13. **(D, E)** Left and middle: Number and strength of intercellular communication networks inferred by calculating the likelihood of communication in GSE148071 and GSE203360 datasets. The thickness of the lines representing strength or number. Right: The communications between osteoclast and tumor cells or immune cells in GSE148071 and GSE203360 datasets.

Next, we explored RAB13 expression at single level by scRNA-seq data in GSE136394 (comprising 6 types of cells and 13 clusters), GSE161277 (comprising 6 types of cells and 11 clusters) and GSE188741 (comprising 7 types of cells and 11 clusters) datasets. Single-cell subgroup analysis isolated different cell populations and RAB13 high-expression cell populations were identified. As shown in **Figures 6A–C** and **Supplementary Figure 2**, RAB13 expression was exclusively prominent in malignant cells and macrophages, indicating that RAB13 may play a crucial role in macrophage infiltration, which warrants further investigation. Finally, we assessed interactions between different cell subpopulations in CRC patients by CellChat and close intercellular communication was observed between macrophage and CRC cells (**Figures 6D, E**).

### 3.5 GSEA and prediction of potentially sensitive drugs

**Figure 7:**
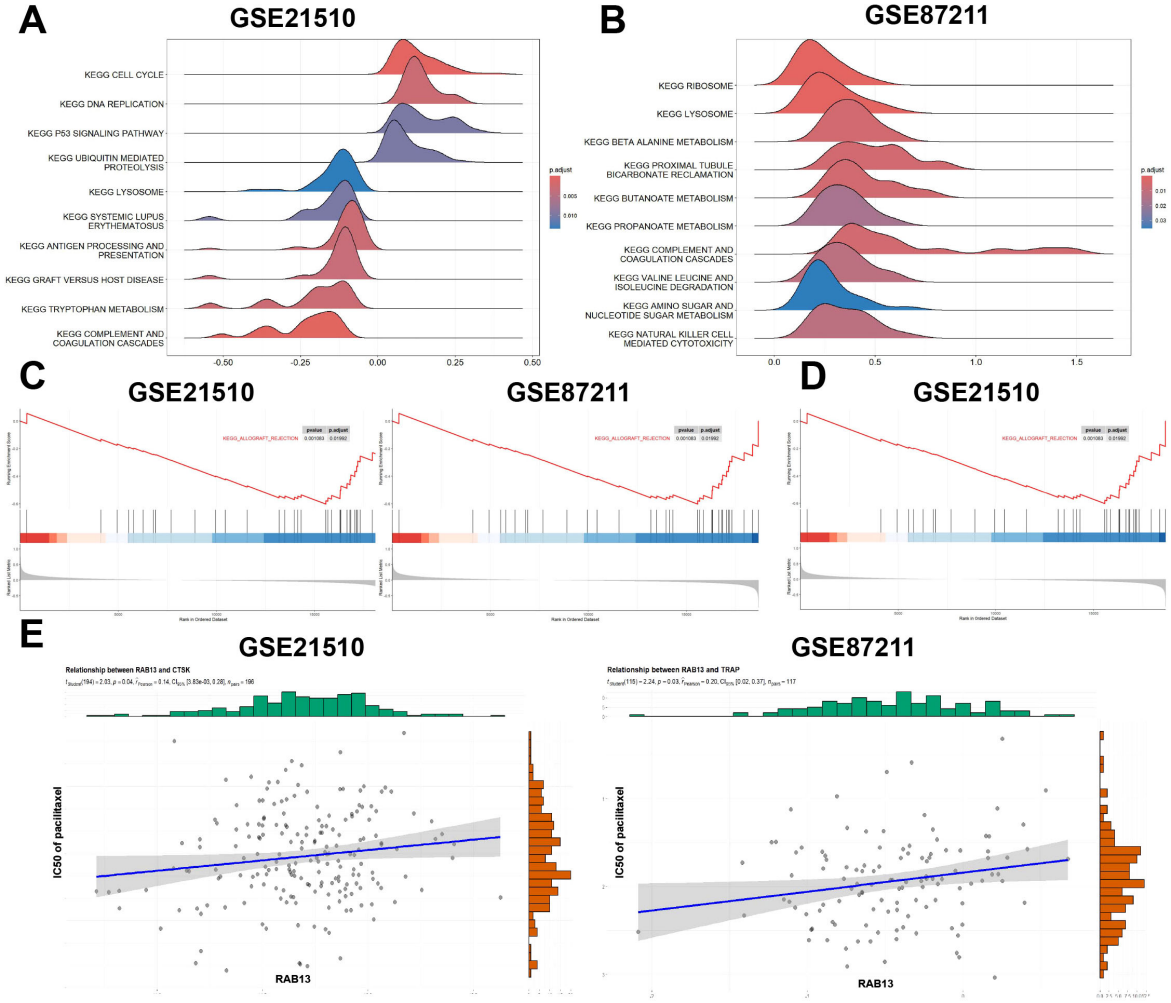
Gene set enrichment analysis (GSEA) and drug sensitivity exploration of RAB13 in COAD. **(A, B)** Enrichment plots from GSEA in GSE21510 **(A)** and GSE87211 **(B)**. **(C)** Enrichment plot of COMPLEMENT_AND_COAGULATION_CASCADES in GSE21510 (left) and GSE87211 (right). **(D)** In GSE21510 dataset, high expression of RAB13 was correlated with CELL CYCLE. **(E)** Pacilitaxel sensitivity prediction with RAB13 in COAD.

In order to obtain understanding into the biological pathways and processes affected by RAB13, GSEA was conducted in GSE21510 and GSE87211 datasets (**Figures 7A, B**). The results showed that COMPLRMENT AND COAGULATION DESCADES was enriched in RAB13 high expression group in the two datasets collectively (GSE21510: NES = -0.60, adjust P = 1.38E-06, **Figure 7C** left; GSE87211: NES = -0.52, adjust P = 1.08E-03, **Figure 7C** right). Coagulant substances such as von Willebrand factor (vWF) are stored in Weibel Palade bodies (WPB), a specialized secretory granules, in a ready-to-be-used form and the biogenesis of WPB are regulated by RAB13 (35). Besides, in GSE21510 datasets, CELL CYCLE was enriched in RAB13 high expression group (**Figure 7D**).

Next, we employed the R package ‘oncoPredict’ to explore the common chemotherapy drugs sensitivity with different RAB13 expression. The results showed that the IC50 values of paclitaxel was positively correlated with RAB13 expression (GSE21510: Pearson’s r = 0.14, P = 0.04, **Figure 7E** left; GSE87211: Pearson’s r = 0.20, P = 0.03, **Figure 7E** right). These results suggested that RAB13 impacted paclitaxel sensitivity in CRC.

## 4 Discussion

Most of CRC patients is died of metastasis, which is developed in nearly half of CRC patients when diagnosed and leading to a poor prognosis (36). Patients have a heterogeneous prognosis, as there are great differences in metastasis patterns and tumor characteristics among CRC (37). Therefore, it is necessary to identify CRC patients with high metastatic risk for treatment decision-making, long-term survival assessment and follow-up frequency. In this study, by employing public datasets, we filtered and identified 74 CRC-PM DEGs, and GO and KEGG analyses were performed on these 74 DEGs.

It is indispensable to find new diagnostic biomarkers and therapeutic targets to improve the prognosis and quality of life of CRC patients. Comprehensive studies have suggested that abnormal gene expression and gene variation may be involved in the colorectal carciongenesis, however, the molecular pathogenesis of CRC is not fully understood. Rab proteins, members of the RAS GTPase superfamily, had crucial roles in the membrane trafficking and fusion events (38). RAB13’s expression has been reported to be aberrant in many cancers, including hepatocellular carcinoma (39) and gastric cancer (40), but there is rare study on the RAB13 expression and clinical significance in CRC patients. Our study found that RAB13 was up-regulated in CRC and high expression of RAB13 indicated shorter progress free survival.

At the genetic level, CRC is a clonal process where somatic gene mutations accumulate to result in abnormal growth of normal intestinal epithelial cells. Tens of drugs targeting receptor tyrosine kinase (RTK) in CRC patients have been uncovered. In this study, we found that druggable mutations, such as EGFR, had higher mutation frequencies in RAB13-low expression group. That indicated CRC patients with low RAB13 expression may get benefit from targeted therapy.

Tumor-infiltrating immune cells were closely related to the tumor biological behavior and prognosis (41). However, the role of tumor-infiltrating immune cells in CRC-PM is still not clear. In our study, by different algorithms, we found RAB13 expression was positively correlated with macrophages, and other immune cells. Zhu et al’s study in breast cancer also showed that RAB13 controlled the C-X-C chemokine receptor type 1/2 (CXCR1/2) membrane translocation to make tumor cells to interact with tumor-associated macrophages (42), indicating RAB13 mediated interaction of tumor cells and macrophages in different cancers. The scRNA-seq technologies dissected the heterogeneity of tumor-infiltrating immune cells and their interactions with the diverse cell types in tumors (43). By scRNA analysis in GEO datasets, we found that RAB13 was observed in tumor cells and macrophages, and cell chat analysis showed that osteoclasts interacted with tumor cells in CRC.

In this study, we showed RAB13’s role and its potential value in CRC-PM, nevertheless, some limitations existed in our research. Firstly, the mechanisms of RAB13 in CRC-PM are necessary to be validated. Secondly, we only used several GEO cohorts for verification, which may have some systematic biases. At last, CRC-PM is a complex process but we only considered the association between RAB13 and CRC-PM, and not consider the relationship between genes and genes or environment (44). We will implement these limitations in future studies.

## 5 Conclusion

In conclusion, this study provided a better understanding of RAB13 in CRC-PM and for further investigation of CRC-PM diagnosis and treatment.

## Data Availability

Publicly available datasets were analyzed in this study. This data can be found here: NCBI GEO database (https://www.ncbi.nlm.nih.gov/gds/); GeneCards database (https://www.genecards.org/); TCGA (https://www.cancer.gov/ccg/research/genome-sequencing/tcga); MethSurv database (https://biit.cs.ut.ee/methsurv); CPTAC database (https://proteomics.cancer.gov/programs/cptac).
