# Supplementary figures and images for: Identification and validation of Rab GTPases RAB13 as biomarkers for peritoneal metastasis and immune cell infiltration in colorectal cancer patients
Source: Front Immunol. 2024 Aug 13;15:1403008. doi: 10.3389/fimmu.2024.1403008 (PMC11347351; doi:10.3389/fimmu.2024.1403008)

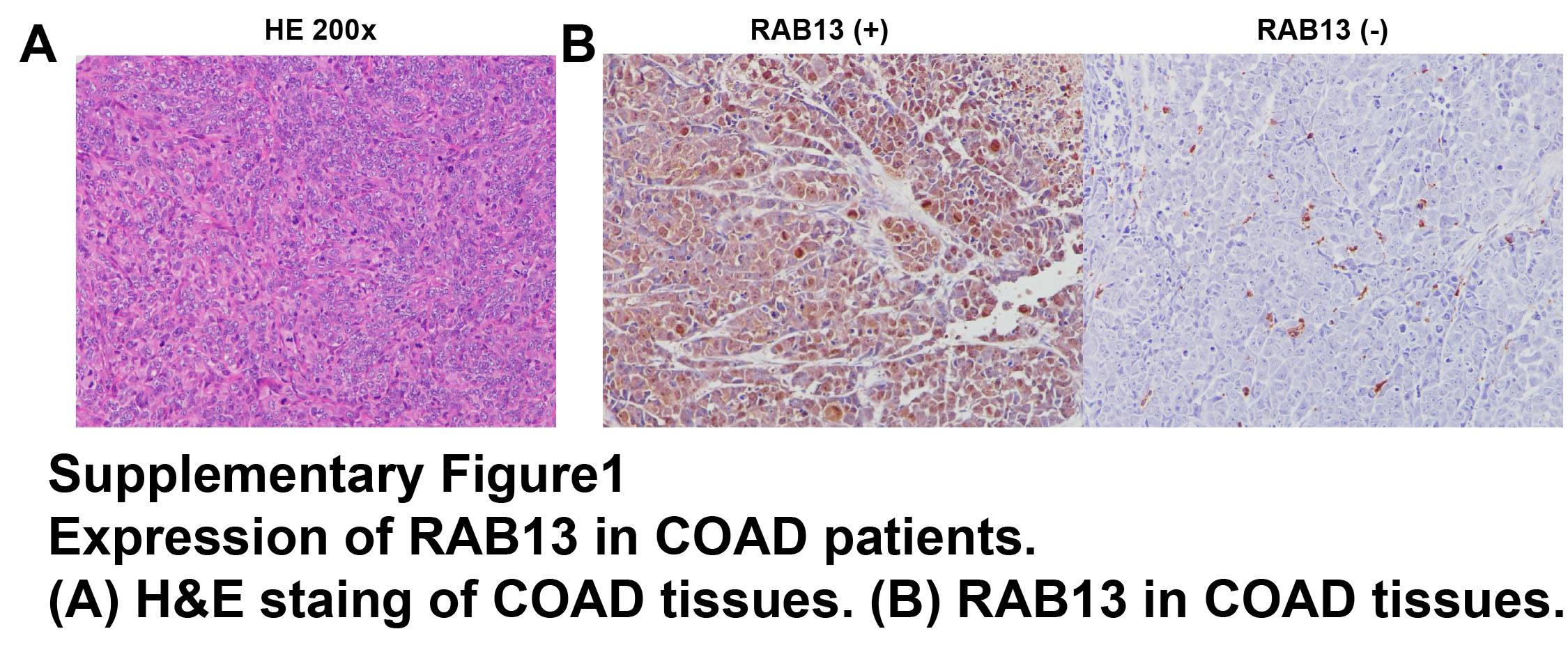

Supplement: Supplementary file 8 [file Image1.jpeg]

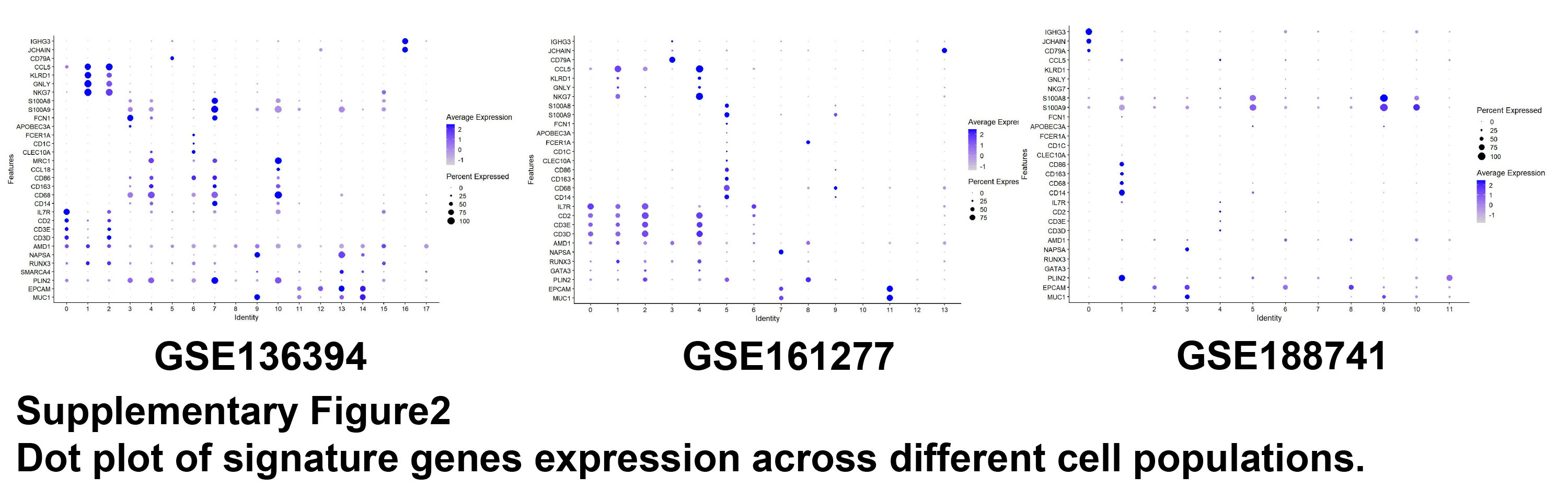

Supplement: Supplementary file 9 [file Image2.jpeg]
