# Supplementary material for: Identification and validation of Rab GTPases RAB13 as biomarkers for peritoneal metastasis and immune cell infiltration in colorectal cancer patients
Source: Front Immunol. 2024 Aug 13;15:1403008. doi: 10.3389/fimmu.2024.1403008 (PMC11347351; doi:10.3389/fimmu.2024.1403008)
Supplement: Supplementary file 10 [file Table1.docx]

**Supplementary Figure1**

161 differentially expressed genes (DEGs) were identified from three datasets.

DEGs Genes

Upregulated *KRT80, MMP7, KRT23, FOXQ1, WNT2, LEMD1, KLHL42, ESM1, INHBA, DPEP1, FABP6, CLDN2, NOTUM, SYK, TCN1, ANXA2, PLAG1, DSG3, FOXC1, CST1, KLK6, IL11, KRT6B, USP11, GRIN2D, CDH3, CLDN1, EPHX4, REG1A, ABCG5, SLCO1B3, CPNE7, LINC00460, FAP, KLK7,* ***RAB13****, SFTA2, ETV4, STRA6, CA9, COL10A1, MMP10, REG1B, ACSL6, SHISA2, ADAMTS12, FUT1, MDFI, CRNDE, COL11A1, OLR1, MSX2, ZIC2, RNF183, S100A2, SLC4A11, HS6ST2, FJX1, REG3A, CST2, TRIM29, DKK4, KRT17, PITX2, MMP3, THBS2, PRSS33, COMP, LRP8, IL1A, APLN, NXPH4, CXCL5, MMP13*

Downregulated *PTGDR, ZG16, TTLL6, CD22, C11orf86, HAPLN1, OSR1, CNTN3, DNASE1L3, CA4, ABI3BP, SCNN1B, ASPG, CLCA4, MS4A12, SLC4A4, CHGA, BEST2, CD177, TRPM6, GREM2, ANGPTL7, PDE6A, SPINK2, AQP8, B3GALT1, CCL23, SCNN1G, CTSG, NAP1L2, FAM107A, CWH43, OGN, TPH1, CDKN2B-AS, LIFR, SCN9A, RSPO2, SCARA5, C16orf89, AMPD1, TEX11, WISP2, ADH1B, GLP2R, KRT24, GUCA2B, BCHE, PRKG2, SLC25A34, CNTFR, TMEM100, C2orf40, INSL5, CA1, BEST4, SPIB, SFRP1, MYOC, ABCA8, CA7, RERGL, SLC6A19, PYY, SLC30A10, SST, TMIGD1, PI16, MAMDC2, SCGN, GCG, CLDN8, OTOP2, UGT2A3, ADH1C, SLC26A3, ADH1A, HEPACAM2, CPB1, SI, CA2, GUCA2A, RFX6, ITLN1, NEUROD1, CLCA1*
